# Chemopreventive effects of the polyunsaturated fatty acids omega-3 on the carcinogenesis process of the upper aerodigestive tract induced by 4-nitroquinoline-1-oxide in Swiss mice

**DOI:** 10.3332/ecancer.2014.392

**Published:** 2014-01-29

**Authors:** Ricardo Ribeiro Gama, Allan Giovanini, Fernanda Scarmato de Rosa, Daniel Cury Ogata, André Luiz Vettore de Oliveira, Ana Flávia Cardoso Costa, Carolina Talini, Denise Feniman, Douglas Kamei, Celso Felipe Júnior, Allan Coco, André Lopes Carvalho

**Affiliations:** 1 Postgraduate Programme in Oncology, Universidade de São Paulo, São Paulo, SP, Brazil and Operative Technique and Experimental Surgery Laboratory, Faculdade Evangélica do Paraná, Alameda Augusto Stelfeld, 2134 Curitiba, PR 80730-150, Brazil; 2 Oral Pathology Laboratory, Dentistry, Universidade Positivo, Curitiba, PR 81200-100, Brazil; 3 Pharmacy and Biochemistry Laboratory, Centro Universitário da Fundação Educacional de Barretos, Barretos, SP 14784-400, Brazil; 4 Research Laboratory, Pathology, Universidade do Vale do Itajaí, Itajaí, SC 88302-901, Brazil; 5 Molecular Biology of Cancer Laboratory, Department of Biological Sciences, Universidade Federal de São Paulo, São Paulo, SP 04021-001, Brazil; 6 Operative Technique and Experimental Surgery Laboratory, Faculdade Evangélica do Paraná, Curitiba, PR 80730-000, Brazil; 7 Head and Neck Oncology Department, Barretos Cancer Hospital, Barretos, SP 14784-400, Brazil

**Keywords:** head and neck cancer, squamous cell carcinoma, 4-NQO, carcinogenesis, chemoprevention, unsaturated fatty acids

## Abstract

**Objective:**

To study the potential chemopreventive effects of polyunsaturated fatty acids omega-3 in Swiss mice submitted to oral and oesophageal carcinogenesis induction by 4-nitroquinoline-1-oxide (4-NQO).

**Study design:**

The animals underwent carcinogenesis induction with 50 µg/mL 4-NQO for eight weeks. The animals were divided into groups: Group I—4-NQO induction without chemoprevention, Group II—chemoprevention with the addition of 5% fish oil (FO) in their diet after 4-NQO carcinogenesis induction, and Group III—chemoprevention with 5% FO in their diet during and after 4-NQO carcinogenesis induction.

**Results:**

The incidence of invasive oral carcinoma was: Group I (72.9%), Group II (84.2%), and Group III (64.7%); *p* = 0.34. The difference in the incidence of invasive oesophageal carcinoma was statistically significant: Group I (37.8%), Group II (68.4%), and Group III (29.4%); *p* = 0.02.

**Conclusions:**

4-NQO induction led to cancer in the majority of animals. Chemoprevention with FO brought no benefit in preventing the carcinogenesis process initiated by 4-NQO for oral cancer. The suggestive pro-tumour action of FO when given after tumour post-initiation seems to demonstrate that this fatty acid can potentialise the action of 4-NQO in the oesophagus carcinogenesis of the Swiss mice.

## Introduction

Head and neck squamous cell carcinoma (HNSCC) is the fifth most common neoplasia in the world [1]. In the United States, it accounts for 3.3% of all malignancies. For 2013, the American Cancer Society estimated approximately 53,640 new cases of upper aerodigestive tract (UADT) cancers—oral cavity, pharynx, and larynx cancers—which resulted in 11,520 deaths [2] approximately. In 2009, the estimate was 500,000 new cases of HNSCC worldwide [1].

Despite the advances in the therapeutics of HNSCC, diagnosis is still quite often done at an advanced clinical stage. In addition, patients have been exposed to the main risk factors (tobacco and alcohol) for a long time, thus the risk of developing a second primary tumour can reach as much as 3–7% per year in these patients [3]. The second UADT primary is considered to be the leading cause of cancer death among patients with initial stage head and neck cancer who had been adequately treated in the past and had a long term follow-up [4].

As with many other epithelial tumours, HNSCC derives from the accumulation of myriad genetic and epigenetic changes occurring in multiple sequential events [5]. The observation of a certain frequency of second primary tumours following a cancer of the oral cavity led Slaughter *et al* [6] to propose the ‘field cancerisation’ theory. This theory suggests that multiple individual primary tumours may arise independently in the UADT as a result of years of chronic exposure to carcinogens. Therefore, it is important not only to treat HNSCC adequately in its first clinical presentation but also to study chemopreventive strategies so that second primary tumours are prevented as they contribute to a major decline in survival of patients with HNSCC in UADT [4, 7].

As defined by Sporn *et al* [8], the term chemoprevention denotes the use of a natural or synthetic chemical agent with the aim of reverting, retarding, or suppressing the progression of carcinogenesis to invasive cancer, or preventing the development of premalignant lesions. Chemoprevention can be provided during the initiation phase inhibiting the development of invasive cancer by blocking the damage to DNA or it can be given during the promotion or progression phases leading to the reversal or suppressing the progression of the premalignant cells, where damage to DNA has already occurred [9].

The nitrous compound 4-nitroquinoline-1-oxide (4-NQO) is a water-soluble quinoline derivative that leads to adducts in DNA molecule as it binds to guanine sites [10] and induces reactive oxygen species production resulting in mutations and DNA-strand breaks [11, 12]—all genetic alterations that are similar to those caused by tobacco carcinogens [13–15]. The carcinogenic effect of 4-NQO was first observed by Nakahara *et al* [16]. This substance has been shown to be effective in inducing squamous cell carcinoma (SCC) of the oral cavity and oesophagus in laboratory animals, such as mice [17–19]. Such induction produces a model of temporal carcinogenesis, which produces multiple preneoplastic, dysplastic, and neoplastic lesions in the same animal after a certain period of chemical exposure [20]. In addition, it does not trigger a local inflammatory reaction and leads to the development of a broad spectrum of lesions that are histopathologically very similar to those seen in the UADT carcinogenesis in humans [21, 22]. Even after the exposure to the chemical has ceased, the carcinogenesis process continues, with the first preneoplastic lesions arising weeks after 4-NQO exposure [7, 22].

Tang *et al* [22] demonstrated that the dilution of 4-NQO in animals drinking water is less laborious and is superior in its carcinogenic induction when compared with the traditional topical exposure, and is sufficient to cause premalignant lesions and invasive SCC of the oral cavity and oesophagus in genetically modified mice. The higher the doses and the longer the period of exposure to the substance in the water, the faster, more efficacious, and more deadly is the UADT carcinogenesis process in the animals.

Saturated fats are implicated in the aetiology of various types of tumours. However, evidence of the anti-tumour action of PUFAS omega-3, such as the eicosapentaenoic acid (EPA) and the docosahexaenoic acid (DHA), is extensively presented in the research literature [23–25]. Polyunsaturated fatty acids (PUFAS) are essential fatty acids obtained from the diet and are subdivided in linoleic acid (omega-6) and α-linolenic acid (omega-3) derivatives. They are key components of cell membrane phospholipids and are substrates to several enzymes [26].

PUFAS omega-6 can be consumed primarily as linoleic acid found in vegetable oils or as arachidonic acid (AA) obtained from meats [27]. PUFAS omega-3 are consumed as α-linolenic acid, and are found in variable concentrations in some oils, such as canola oil and in darkleafed vegetables [26]. Long-chain PUFAS, especially EPA and DHA, are found in some cold-water fishes and in fish oils (FOs) [26].

One of the most important functions of PUFAS is related to its enzymatic conversion into eicosanoids, which are lipids with hormone-like action [28]. Eicosanoids modulate both inflammatory and immune responses, platelet aggregation, cellular growth, and cell differentiation [28]. The eicosanoids production from PUFAS depends on the action of cyclooxygenases (COX) and lipoxygenases (LOX). The COX, acting on AA and EPA, originate prostaglandins and thromboxanes, while the LOX, acting on the same substrates give rise to leukotrienes and lipoxins [26, 28]. The 2-series prostaglandins and thromboxanes and the 4-series leukotrienes, derived from AA, tend to be more pro-inflammatory and pro-proliferative in most tissues, whereas the 3-series prostaglandins and thromboxanes and 5-series leukotrienes derived from EPA are less contributive to inflammation and proliferation [26, 28]. Therefore, EPA-derived eicosanoids are less favourable to tumour cells growth [26] and when EPA/DHA are present and available they will be preferably used by COX and LOX [26, 28]. In conclusion, the supposed chief mechanism through which PUFAS omega-3 reduces the risk of cancer is leading to suppression of pro-proliferative AA-derived eicosanoids biosynthesis [26, 28].

The main objective of the present study was to evaluate the effects of PUFAS omega-3 or FO as a potential chemopreventive agent to prevent the UADT carcinogenesis initiated by 4-NQO in Swiss mice.

## Materials and methods

All experimental work was previously approved by the Research Ethics Committees of the Faculdade Evangélica do Paraná and Universidade de São Paulo and complied with the guidelines for animal handling and care established by the Brazilian College of Animal Experimentation.

The study animals were albino Swiss mice (*Mus musculus*) aged six to nine weeks, with an equal proportion of males and females across different groups. They were bred in an adequate facility with a controlled light-dark cycle (12–14 h/day), temperature (20–23 °C) and air humidity (40–60%). Water and food were given *ad libitum*. Standard isocaloric food for mice was provided for all groups: Nuvilab CR-1 (Nuvital, Brazil).

The carcinogen 4-NQO (Sigma–Aldrich, United States) was homogenised on a weekly basis in propylene glycol to a final concentration of 5 µg/mL. The resulting solution was stored in a refrigerator at 4 °C at the Biochemistry Laboratory, Faculdade Evangélica do Paraná. This solution was further diluted in the drinking water of the mice to a final concentration of 50 µg/mL and changed every 48–72 h for eight weeks. Since 4-NQO is light-sensitive, the bottles were wrapped in aluminium foil to protect them properly from the light in the breeding facility.

Omega-3 was prepared at a 5% concentration mixed into the mashed food by a pharmacist. The capsules of 1 g of omega-3 contained 12.5% DHA (125 mg); 17.5% EPA (175 mg), and 10 mg vitamin E (α-tocopherol). The feedings were prepared on a weekly basis at the Biochemistry Laboratory, Faculdade Evangélica do Paraná and stored at a 1.7–3 °C refrigerator temperature. The recipe was created by the Pharmacy and Biochemistry Laboratory, Centro Universitário da Fundação Educacional de Barretos and its stability at refrigerator and at breeding facility temperature and the efficacy of FO after mixed with the agglutinative agents were tested and approved before giving it to animals. The mixture of PUFA omega-3 to the regular diet was done through the following steps:

(a) Twenty-five grams of corn starch was mixed in 225 mL of water. The ingredients were mixed while being heated until a consistent product was obtained (starch glue);
(b) Three hundred grams of bran (mashed) feed were mixed with 15 capsules of omega-3 (1 g each) plus 0.7g of anti-mold Mix® (2.5%)—5% final concentration of FO;
(c) In a big bowl, the 300 g of bran feed + 5% omega-3 + anti-mold were manually mixed with the agglutinative agents: 40 g of starch glue and with 110 g of gelatin (Mix®);
(d) the final product was solid and could be perfectly filled in a plastic tray with round empty spaces, which were then compressed digitally and let it air dry for 2 h. Finally, they were taken out from the moulds and given to the animals that could gnaw them. The food was replenished every day at the breeding facility.

### Study groups

**Figure 1.**
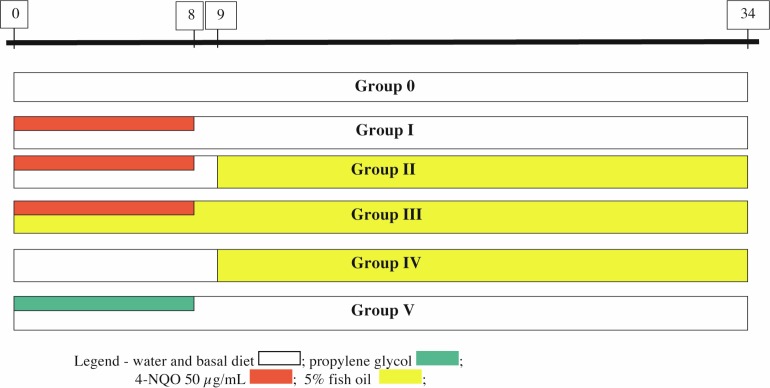
Diagram of the model for tumour induction and chemoprevention in the groups of Swiss mice (timeline in weeks).

The animals were allocated into:

(a) ** Group 0—Control (10 animals):** received only water and food (*breeding facility monitor*);
(b) ** Group I—4-NQO 50 (43 animals):** received 50 µg/mL 4-NQO for eight weeks and no chemoprevention. The group was followed for another 24 weeks;
(c) ** Group II—4-NQO 50 → FO 5% (20 animals):** received 50 µg/mL 4-NQO for eight weeks. One week after tumour induction was concluded, this group received chemoprevention with FO (addition of 5% in the diet) for 24 weeks;
(d) ** Group III—4-NQO 50 + FO 5% → FO 5% (18 animals):** received 50 µg/mL 4-NQO for eight weeks concomitantly with chemoprevention with FO (addition of 5% in the diet). Once tumour induction was finished, chemoprevention continued for another 24 weeks;
(e) ** Group IV—FO 5% (5 animals):** received an addition of 5% FO in the diet for 24 weeks;
(f) ** Group V—Propylene glycol (10 animals):** received only vehicle (**propylene glycol**) for eight weeks and was followed for another 24 weeks. (Figure 1)

### Necropsy and histological study

The study was concluded 34 weeks after its start.

The animals were sacrificed in a CO_2_ chamber at the end of the study or when they exhibited signals of cachexia, lethargy, or agony by tumour progression. At one of these moments, the animals were necropsied and the organs of interest were removed at Operative Technique and Experimental Surgery Laboratory, Faculdade Evangélica do Paraná, photographed and referred to the Pathology Laboratories.

The oral cavity specimens were sent properly fixed and immersed in formalin 10% overnight, and then finally analysed by one experienced oral pathologist at the Oral Pathology Laboratory, Dentistry, Universidade Positivo. The oesophageal specimens were sent properly opened, fixed, and immersed in formalin 10% overnight, and then finally analysed by another experienced digestive pathologist at the Research Laboratory, Pathology, Universidade do Vale do Itajaí. The macroscopic description of the lesions were done and recorded by the pathologists. Cross sections of the tissues and tumours were prepared and after this they were embedded in paraffin and sectioned into 5-µm sections. The sections from the tongues and oesophagi were deparaffinised, rehydrated, and stained with H&E for histopathology.

The oral cavity lesions were classified as: (1) epithelial hyperplasia and hyperkeratosis, (2) slight to moderate dysplasia, (3) carcinoma *in situ*, (4) verrucous carcinoma, and (5) invasive SCC [29, 30].

The oesophageal lesions were classified as: (1) epithelial hyperplasia and hyperkeratosis, (2) low-grade dysplasia, (3) high-grade dysplasia, (4) carcinoma *in situ*, (5) verrucous carcinoma, and (6) invasive SCC [31, 32].

Hyperplasia was defined as thickened epithelium with prominent surface keratinisation and with or without elongated rete ridges. Dysplasia was defined as loss of polarity in the epithelial cells, nuclear pleomorphism and hyperchromasia, abnormal single cell keratinisation (dyskeratosis), and increased or abnormal mitoses. Lesions with such changes involving the entire thickness of epithelium were considered as carcinoma *in situ*. Verrucous carcinoma was defined as noninvasive exophytic growth of neoplastic cells (verrucous aspect), and invasive SCC was defined as a lesion with invasion into the subepithelial tissues [22, 32].

### Statistical analysis

For the statistical analysis, the SPSS 18.0 software was used. Chi-square or Fisher’s exact test were used to assess the differences in the incidence rates of the tumour lesions found across the different groups in the study. For the survival analysis, the nonparametric Kaplan–Meier estimator was used, while the *log rank test* was used to compare survival curves. For all statistical tests, *p*-values lower than 0.05 (5%) were considered statistically significant.

## Results

Incidence of invasive oral and oesophageal carcinomas and the effects of chemoprevention

Six animals from group I, one from group II, and one from group III died early (earlier than 20 weeks after tumour induction was concluded) and were excluded from the statistical analysis of invasive neoplasia incidence. The fact that this exclusion was done is based in literature on the subject that has established 20 weeks as the minimum period of time that invasive SCC can be detected in UADT [22].

Of all the analysed animals exposed to the action of 4-NQO, 90.5% developed invasive neoplasia in at least one UADT organ. The remaining 9.5% did not; however, they already showed preneoplastic lesions. The incidence rate of oral carcinoma in group I was 72.9%, while 37.8% was the rate for oesophageal carcinoma. If the same animal had premalignant and malignant lesions in the same organ only the malignant (invasive) was computed.

At the end of the period, when all animals were euthanised, the incidence for oral neoplasia was 72.9% in group I, 84.2% in group II, and 64.7% in group III. The intergroup comparisons yielded no statistically significant difference in the incidence of invasive neoplasia of the oral cavity (*p* = 0.34) (Table 1).

Regarding the induced oesophageal neoplasias the authors observed a higher incidence rate of invasive oesophageal carcinomas in group II (68.4%) when compared with group I (37.8%); *p* = 0.015. It was seen also when comparing group II with group III (29.4%); *p* = 0.021. There was no statistical difference between groups I and III (*p* = 0.71) (Table 2).

### Survival analysis

Over the weeks that followed the conclusion of tumour induction, there was a considerable loss of animals as a result of the natural course of the carcinogenesis process as well as from other causes, such as bronchopneumonia, action of 4-NQO/FO, and indeterminate causes. During the observation period in group I, five animals died due to cancer and nine animals due to other causes, such as infection, tissue damage, and organic failure caused by 4-NQO action. During the chemoprevention period, six animals died due to cancer and three due to other causes in group II, while in group III there were two and four deaths, respectively.

Among the 4-NQO 50 groups that received chemoprevention or not, there was no statistically significant difference in mortality rates: between groups I and II (*p* = 0.07), between groups I and III (*p* = 1.00), and between the groups II and III (*p* = 0.23).

**Figure 2.**
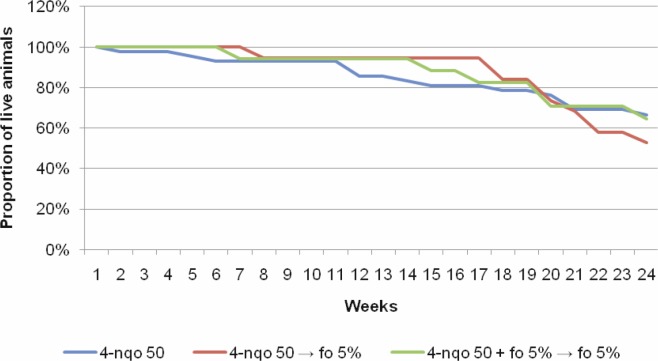
Overall survival over the 24 weeks following the conclusion of tumour induction (8 weeks) across the groups of interest.

The analysis of the overall survival over the 24 weeks after the period of tumour induction with 4-NQO 50 µg/mL, followed or not by chemoprevention with FO at different phases of carcinogenesis did not show a statistically significant difference across groups (*p* = 0.74) (Figure 2).

## Discussion

In the present study, chemically induced UADT carcinogenesis was successfully obtained in Swiss mice trough 4-NQO diluted in mice drinking water for eight weeks.

While there are reports on the use of other carcinogenic agents, such as benzopyrene in these animals [33, 34], no report exists in the literature concerning the induction of UADT carcinogenesis in Swiss mice after 4-NQO exposure. The animals in groups I, II, and III were observed for another 24 weeks, totalling 32–34 weeks of experiment duration, per group. As a result, an incidence rate of 72.9% for oral neoplasia was found in group I, while 37.8% was the rate for oesophageal carcinoma. In addition, many animals developed an association of invasive neoplasms in more than one organ. This raises the incidence of neoplasms per group. Therefore, in group I, the authors found at least one UADT invasive carcinoma in 83.7% of the animals.

The exclusion of eight animals who died early on the study, considering the observation that the vast majority of animals in the present study showed invasive lesions after 20 weeks should not affect the observed results. Therefore, there is a low likelihood of underestimating tumour induction on account of early deaths from other causes than tumour-related ones since the length of time might not have been sufficient for the development of invasive lesions in group I [22]. In addition, in groups with chemoprevention, the possibility of overestimating is excluded, by minimising the chance of mistakenly concluding that the animals were protected from cancer by chemoprevention efficacy when in fact they did not develop it because they died or were sacrificed early, before carcinogenesis reached the invasive stage.

Tang *et al* [22] administered 4-NQO in the water of CBA and C57BL/6 mices for 8 or 16 weeks at concentrations of 20, 50, and 100 µg/ mL and observed the animals for another 16 and 8 weeks, respectively (total duration of the experiment: 22–24 weeks). There was 100% incidence of SCC of the tongue, 16 weeks after the conclusion of tumour induction, in mice exposed for eight weeks to 50 µg/mL 4-NQO. A lower rate of oesophageal neoplasia was found (33%).

When the two studies are compared it can be observed that although the incidence of oesophageal neoplasia is higher in the present study the incidence of oral neoplasia is not. The reason for that difference is not clear since the animals in the present study were observed for a longer time and it is known that the longer the observation period the higher the incidence of invasive neoplasms observed at the end of the experiment. One of the reasons may be the different strains of animals used since the mice in the study by Tang *et al* [22] were genetically manipulated to express modified versions of relevant genes which may facilitate the action of 4-NQO in the initiation stage and even accelerate the carcinogenesis process on account of a previous genetic damage (susceptible area) associated with a carcinogenic agent exposure.

The addition of PUFAS omega-3 to the diet is known to induce pro-differentiation, anti-proliferation, and pro-apoptotic mechanisms in various types of cancer that have been studied [28]. However, epidemiological studies seeking to correlate the intake of PUFAS omega-3 and the risk of cancer in humans are inconclusive [35]. Approximately half of the studies attempting to correlate the increase in PUFAS omega-3 intake with a lower risk of cancer failed to demonstrate that association [28]. One of the plausible explanations for this negative finding is that the population who used PUFAS omega-3 did so at doses that were too small to produce any protective effect [28], as opposed to the studies conducted with animals, in which the very high doses led to positive results described in literature on the subject [36].

There is no consensus regarding the anti-tumour dose of PUFAS omega-3. The studies mention addition of 10%, 20%, or even 30% FO in animals laboratory diet, with positive results in cachexia control, in immune system stimulation, and even in tumour growth inhibition in some neoplastic cell lines [36]. These doses, if administered to humans, are much greater than the doses conventionally used for a hypolipemic effect. The feasibility of using it in humans would require testing.

In the current study, FO was not helpful in arresting the carcinogenesis process initiated by 4-NQO. Both the incidence rate of oral neoplasia and that for invasive oesophageal neoplasia were greater for group II. With regard to the oesophagus, this fact is corroborated by the significant statistical difference in the comparison with other groups. The literature describes an anti-tumour action of a given agent on an organ or tissue and the possible pro-tumour action of the same agent on another organ or tissue, as observed by Mayne *et al* [37] with β-carotene, which showed promise in the chemoprevention of HNSCC, however revealed a potential pro-tumour effect on the respiratory tract and lungs on smokers. Based on our results, we believe that the use of FO on post-initiation (group II) may have somehow potentialised the oesophageal carcinogenesis initiated by the action of 4-NQO.

## Conclusion

The model of UADT carcinogenesis induced by 4-NQO in Swiss mice is effective. However, based on the methodology employed in the present study and the results obtained, the use of the different forms of chemoprevention with PUFAS omega-3 (FO) was not beneficial in preventing, retarding, or suppressing the carcinogenesis of the UADT induced by 4-NQO.

Interestingly, the suggestive pro-tumour action of FO when given after tumour post-initiation seems to demonstrate that this fatty acid can potentialise the action of 4-NQO in the oesophagus carcinogenesis of the Swiss mice. This finding requires further research and validation by other studies.

## Figures and Tables

**Table 1.** Incidence of oral cancer among the groups.

Groups (*n*)	Invasive oral SCC		
	Present *n* (%)	Absent *n* (%)	*p*-value
4-NQO 50 (37)	27 (72.9)	10 (27.1)	0.34
4-NQO 50 → FO 5% (19)	16 (84.2)	3 (15.8)	
4-NQO 50 + FO 5% → FO 5% (17)	11 (64.7)	6 (35.3)	

SCC: squamous cell carcinoma; FO: fish oil

**Table 2.** Incidence of esophageal cancer among the groups.

Groups (*n*)	Invasive SCC in esophagus		
	Present *n* (%)	Absent *n* (%)	*p*-value
4-NQO 50 (37)	14 (37.8)	23 (62.2)	0.02
4-NQO 50 → FO 5% (19)	13 (68.4)	6 (31.6)	
4-NQO 50 + FO 5% → FO 5% (17)	5 (29.4)	12 (70.6)	

SCC: squamous cell carcinoma; FO: fish oil
